# Varieties of improvement expertise: Knowledge and contestation in health‐care improvement

**DOI:** 10.1111/1467-9566.13616

**Published:** 2023-01-27

**Authors:** Alan Cribb, Vikki Entwistle, Polly Mitchell

**Affiliations:** ^1^ Centre for Public Policy Research King’s College London London UK; ^2^ Health Services Research Unit and School of Divinity, History and Philosophy University of Aberdeen Aberdeen UK

**Keywords:** expertise, health service organisations, norms and values, quality of care, research and policy, sociology of scientific knowledge

## Abstract

The ‘improvement’ of health care is now established and growing as a field of research and practice. This article, based on qualitative data from interviews with 21 senior leaders in this field, analyses the growth of improvement expertise as not simply an expansion but also a multiplication of ‘ways of knowing’. It illustrates how health‐care improvement is an area where contests about relevant kinds of knowledge, approaches and purposes proliferate and intersect. One dimension of this story relates to the increasing relevance of sociological expertise—both as a disciplinary contributor to this arena of research and practice and as a spur to reflexive critique. The analysis highlights the threat of persistent hierarchies within improvement expertise reproducing and amplifying restricted conceptions of both improvement and ‘better’ health care.

## INTRODUCTION

Health‐care improvement matters because health care frequently falls short of reasonable expectations, and this has crucial, sometimes life‐changing, implications for both patients and health professionals. And it has long been understood that this need not be seen as a comment on the shortcomings of individuals but rather requires attention to the design, organisation and cultures of health‐care systems that can be more or less successful in enabling good‐quality care. The existence of forms of expertise about health‐care improvement is thus important. However, deciding what counts as appropriate forms of expertise is not straightforward. Someone coming anew to the field will learn about ideas and practices that have emerged over a very long period but which, from the late 1990s, coalesced into the notion that improvement should aspire to be and could be labelled as a ‘science’. Yet, over the same period, approaches to knowledge have become plural, decentralised and to some extent democratised in a ‘knowledge agora’ where competing claims to expertise jostle with one another (Nowotny, 2003).

This article draws on and hopes to contribute to both the sociology of expertise and the sociology of improvement. Sociological analyses of expertise show how it does not simply reside ‘in the heads’ of specialist scientists or professionals but is embedded in material and institutional contexts and sets of practices, and across distributed networks of knowledge generation (Knorr‐Cetina, 2007). In addition, for it to serve its function ‘expertise must be able to understand the inter‐linkages that bind diverse practices, institutions and networks of diverse actors together’ (Nowotny, 2003, p. 152). This means redrawing (fuzzier) boundaries around expert knowledge and rethinking the mechanisms of cooperation and co‐option that underpin the power of expertise (Eyal, 2013). In what follows, we set health‐care improvement within this frame, exploring the construction of improvement expertise and the tensions that arise from it both coming to be represented as a science and simultaneously becoming more epistemologically expansive and contested.

Sociological contributions to health‐care improvement have become a key part of the epistemological broadening of the field. Work in sociology makes a direct contribution to improvement research but also embodies and enables a ‘standing back’ by illuminating the social contexts and conditions of both knowledge production and improvement efforts. Writing in 2016 about the growing role of sociology in the study of health‐care quality and safety, Waring et al. distinguish sociology from the ‘dominant disciplines’—medical science, social psychology, human factors, ergonomics and safety science—arguing that whilst these disciplines:

have done much to advance understanding of the sources of quality and safety within the clinical micro system and work environment, sociology can build on this perspective to furnish insights into the cultural, socio‐technical, political and institutional forces that influence care quality.

(2016, p. 199)

The rise of improvement as a ‘science’ marks a shift from seeing ‘improvement expertise’ largely as practical know how—based on sets of methods developed, for the most part, from industrial quality control and improvement, to expertise which consciously combines practical and theoretical knowledge and which enjoys both academic and extra‐academic standing. ‘Improvement science’ involves the idea that local practical knowledge can be both harnessed and complemented by the use of more systematic and rigorous research methods, supported by broader epistemological and theoretical bases, with a view towards constructing a body of improvement knowledge with wide applicability (Marshall et al., 2013). Over a similar period but from different origins, the closely related field of ‘implementation science’ came into being. This was part of the broader ‘evidence‐based’ health‐care movement and is focussed on the scientific study of the uptake of best evidenced health‐care practices—that is, it seeks to combine research, theory and practice to close the ‘implementation gap’ between what the research base identifies as good‐quality practice, especially ‘effective practice’, and what happens on the ground. These fields or sub‐fields are inevitably shaped by their intellectual and practical origins. In the case of implementation science, this includes, for example, the legacy of studies on the ‘diffusion of innovations’ and ‘knowledge utilisation’, and in improvement science the ongoing influence of industrial management including the management of process variation through PDSA (Plan‐Do‐Study‐Act) cycles (Nilsen et al., 2022). Likewise, both of these fields have grown by drawing on ever broader disciplinary and inter‐disciplinary resources and thereby gradually taken on a more eclectic character, and this has recently included recognition of and repeated calls for convergence or at least cross‐fertilisation between them (Nilsen et al., 2022; Øvretveit et al., 2021). The framing of expertise as ‘science’, including the emphasis on rigour and generalisability, suggests a knowledge hierarchy in which some people are producing the ‘best knowledge’ on behalf of others; yet the growing emphasis on breadth and eclecticism suggests a more inclusive conception of diverse knowledge(s) (Gaventa & Bivens, 2014).

Improvement expertise thus provides a helpful case to analyse the relationships between knowledge hierarchy and inclusiveness. The notion that certain ‘ways of knowing’ are systematically devalued in health care is much discussed, most obviously in problematisations of evidence‐based medicine’s ‘evidence pyramid’ (Timmermans & Berg, 2010). The need to combine different kinds of knowledge to inform practical decision‐making has led some to explicitly call for models that are ‘horizontal’ rather than ‘vertical’ (Trnka & Stöckelová, 2019). Analogous calls have been made in the name of ‘epistemic justice’ (Fricker, 2007). This requires not only changes in interpersonal relationships in which credibility is granted to more voices (‘testimonial justice’) but changes to the structural conditions of knowledge production so that more groups have a role in producing the frameworks and lenses through which we collectively make sense of the world (‘hermeneutic justice’). However, work on democratising currents show that there is no clear‐cut split between vertical and horizontal tendencies. The ‘boundary work’ (Gieryn, 1983) that sustains hierarchy exists alongside the potential for boundaries to positively link together ways of knowing, thereby connecting different constituencies and perspectives (Cramer et al., 2018). For example, research on Environmental Impact Assessment (Barnard‐Chumik et al., 2022) has highlighted genuine pluralism in which the ‘knowledge construction process’ displays ‘horizontality’ including an emphasis on integrating multiple ways of knowing and, at the same time, the maintenance of knowledge conflicts. This work points to ‘mechanisms of power’ that reproduce epistemic authority despite bridge building and integration. Improvement expertise is, we suggest, a useful focus for further exploring these two interacting tendencies and one that can also be used to help specify the mechanisms that reproduce hierarchy and their effects on health policy discourses.

## METHODS

This article is based on semi‐structured interviews with 21 senior leaders in UK and global health‐care improvement. The topic guide was developed by the researchers through several iterations (including at two points in the interview process as new issues emerged) as part of a broader study of foundational issues in health‐care improvement and was designed to elicit wide‐ranging insider reflections on: (a) the relationship between their biography and the field of health‐care improvement (b) what they saw as the ‘state‐of‐the‐art’ questions and challenges in the field, including more specifically (c) their perspectives on conceptual and ethical issues in improvement.

As a starting point, we identified people who would be expected to feature at plenaries in national and international health‐care improvement conferences and as editorial authors. We used purposive sampling to achieve balance in relation to gender (based on published pronouns) and to represent minoritised ethnic identities (relative to a UK context) as well as to reflect different ‘sectors’. The vast majority of people we approached agreed to be interviewed, whilst a few declined, mainly citing heavy work commitments (typically linked to pandemic adjustments). Participants often worked across academic and policy and/or practice contexts and several were engaged in ‘brokering’ inter‐sectoral relationships, but we aimed to achieve some variation in principal institutional homes (with just under half being based in university roles and just over half in service, policy or advisory roles). We also ensured that we invited people with reputations in different areas of health‐care improvement (e.g. safety, person‐centred care etc.). All the participants had been working on these issues for at least 15 years (and typically many more) and had a high‐profile role in research and policy/practice communities and had helped frame academic and policy debates along with education and training. For the most part, we construed the field of health‐care improvement in a comparatively circumscribed way to pick out people who worked in ‘Quality Improvement’, ‘Improvement Science’, and ‘Implementation Science’ (or similar e.g. ‘Improvement Studies’) but we also included several people who were routinely treated as influential figures in these areas but whose reputations (e.g. in Health Services Research and/or Patient Advocacy) preceded the full establishment of these sub‐fields and, in some cases, who resisted ‘improvement’ or ‘implementation’ related labels. Given their seniority and influence, the sample had a very strong international profile although most of the participants were UK‐based (because the sampling was done when the interviews were planned as face to face encounters). However, enabled by the shift to online working, we also included six prominent non‐UK figures drawn mostly from North America and Australia.

Interviews averaged an hour in length. They were conducted conversationally, supported by the topic guide with some suggested prompts to open discussion of potential areas of interest. They were digitally recorded and professionally transcribed verbatim. All three authors familiarised themselves with the data and read and coded transcripts working collaboratively to develop a thematic framework (Pope et al., 2000). The analysis drew upon the constant comparative method to identify and consolidate themes (Glaser, 1965). In addition to this inductive approach, as the analysis progressed, senstisation to themes was increased through dialogue with existing literature in the sociology of expertise and improvement. Progressive iterations of themes were developed through team discussion of memos and summaries. The unanticipated theme of ‘contested knowledges’ emerged very strongly and clearly during the first phase of the analysis, and, subsequently, sub‐themes developed relating to role positions, science/epistemology and reflexivity/scepticism. The three authors shared that the interviewing between them and rigour was enhanced by all the authors working across the full dataset and jointly participating in the thematic analysis alongside ongoing dialogue with each other and with collaborators in the field over a 2 year period of engagement in improvement‐related projects as part of the broader study (Jensen, 2008). As health‐care improvement remains a comparatively small field, avoiding personal identification of these high‐profile participants is challenging. For the most part, we will refer to them simply as ‘participants’ and number them as Participant 1 etc. In a few places where it seems necessary to provide a little context about the speaker, we will do so, but we have deliberately chosen not to provide other contextual material that might have been interesting to the reader and excluded any data extracts that mention examples (or other specifics) that could be identifying.

## FINDINGS

Participants regularly invoked contrasts that displayed or referred to ‘perspectival’ differences. That is, their accounts showed that, and how, the way expertise is constructed—including the kinds of knowledge this makes salient—is partly a function of people’s vantage points and social and epistemic trajectories. In the sub‐sections below, we analyse the way such perspectival differences are manifest and in how participants locate themselves within the ‘knowledge agora’ of improvement. In the first sub‐section, this simply relates to participants’ relationships with academic and/or health systems and practices and the various role positions they occupy. In the second sub‐section, we look at how such contrasts are multiplied within applied improvement research, where role positions and practical and epistemic orientations intersect. Finally, in the third sub‐section, we illustrate and discuss ‘second order’ accounts of expertise—that is, accounts that explicitly ‘stand back’ and reflect on the nature and limits of improvement knowledge—especially currents of scepticism and critical reflexivity. The findings illustrate the potential for improvement expertise to reproduce and amplify restricted conceptions of both improvement and ‘better’ health care. This is further elaborated in the Discussion section, which also highlights how epistemological hierarchies can persist alongside greater diffusion and decentralisation of knowledge(s) (Brosnan & Kirby, 2016).

### Between the academy and practice; between systems and people

The data highlight that health‐care improvement operates at the intersection of various social fields and faces multiple constituencies. Most obviously, improvement experts can see themselves as serving both academic and practice audiences; in the latter case, see their expertise as directed towards serving both the needs of health systems and those of communities. In broad terms, these may all be compatible but, at the least, these constituencies suggest different possible emphases of orientation (Currie et al., 2013). Participants frequently situated their accounts with these contrasts in the background. The questions about expertise raised by their accounts often parallel those previously analysed in sociology of health: that is, questions about the relationships between, on the one hand, official and formal systems of propositional and theoretical knowledge and, on the other hand, ‘experiential’ and ‘tacit’ knowledge derived from immersion in practical activities by both service providers and service users (Maslen & Lupton, 2019; Oborn et al., 2019).

Some participants questioned how far the very existence of a research speciality of ‘improvement’ is desirable given that it risks needless abstraction from practice in all its diversity. A few of these were participants who stressed that improvement should first and foremost be seen as intrinsic to health‐care professional practice:

Don Berwick said, “We all have two jobs: to do our work and to improve our work.” So, I see quality and quality improvement should be just natural parts of our work. The difficulty is when people … when we over‐describe it, and make it into an entity, it becomes the other. And when something becomes the other it becomes exclusive.

(Participant 18)

we have made healthcare work very complex, and we have progressively engaged in that work in contexts of large systems, the net effect of which is to diminish agency by the professional for the ongoing improvement of their work … And that is the nub of the problem.

(Participant 3)

Others, speaking as representatives of patient organisations and voices had reservations about what they saw as the narrowness and exclusionary effects of technical sounding improvement discourses:

I think, yes, we [patient organisations] are improvers but we are not improvers in the way that, you know, the nerds describe it.

that doesn’t mean that they [patient organisations] don’t … work as hard as they can to improve it, but they do so through a very different mix of approaches than the quite narrowly‐defined approaches that seem to kind of characterise what improvement is …

(Participant 13)

I don’t really identify with the language of health improvement. I know it’s now a term with its own meaning, domain, experts and all the rest of it.

(Participant 14)

All the participants had links with both practice and research but some of them identified with particular academic research communities and in some cases presented themselves as operating ‘outside’ improvement practice, albeit aiming to inform it. Others stressed their immersion in improvement practices:

I’m trying to generate knowledge that will help implementation practice but I’m not taking responsibility for implementation in a service setting, because other people can do that. I clearly work with them because when we’re doing research, you know, it has to be embedded in their world. … I’m doing this in … a research perspective, you know, trying to generate generalisable knowledge.

(Participant 12)

I do do improvement … in a very hands‐on, applied, experiential way we will teach them … and mentor them and advise them. So, in that sense, yeah, I am an improvement science practitioner.

(Participant 6)

Participants sometimes emphasised the potential gaps and tensions between academic and practice‐based ways of knowing:

there’s no doubt that there are tribes … between practitioner ownership of improvement science and academic ownership of improvement science, with the academics criticising the practitioner tribe for being insufficiently rigorous and thoughtful and critical and the practitioner improvement scientists being critical of the academics for being narrow in their thinking about what science is and what knowledge is.

(Participant 5)

And I think there is, there is definitely a tension, I think, between that body of work, which is very close to practice, and then, if you like, the much more … the more academic end of improvement science, looking down on that work.

(Participant 6)

In addition to the academic‐practice tension participants sometimes made reference to a distinction between system‐serving and people‐serving. Two of the most widespread negative contrasts deployed by participants refer to conceptions of improvement that are heavily managerial and—overlapping with these—those that fail to be responsive to the needs of service users (even if only, sometimes, as these are construed by clinical professionals). Such comments were often aligned to a recognition that management agendas could be very important for, and contribute to, improvements although the aspects of improvement they prioritised were typically institutionally defined. For that reason, these could easily be in tension with improvement as understood from other vantage points and ways of knowing including clinical vantage points:

what I’ve noticed more is that it’s quite hard to get the managers to take what the clinicians want to do seriously. And I don’t say that as a simple criticism of managers, but their preoccupations are getting stuff done day‐to‐day and, you know, balancing the books and making sure that, you know, this is the right throughput, and improving the actual quality of the service delivered is, in a way, secondary.

(Participant 19)

then managers without an expertise in the specific subject domain, like clinical medicine, find it very difficult to actually manage the quality of what they’re doing …

clinicians own that little piece of esoteric, expert space. But actually it’s so interdependent on all the bits around it … good facilities, good estates, good rooms, …

this bit of quality, or whatever you want to define it, is mine, and I improve this and you improve that.

(Participant 2)

These contrasts between management and clinical perspectives illustrate the ways in which different ways of knowing embody different constructions of, and linkages between, ‘knowledge subjects’, ‘knowledge objects’ and improvement purposes (Meyer & Molyneux‐Hodgson, 2010; Mol & Law, 2004). Apart from a few straightforwardly critical remarks directed at managers pursuing ‘fads’ or ‘easy fixes’, an underlying concern was that managerial conceptions of improvement could be partial and, in particular, miss ‘what matters to people’, including patients. Participants expressing these concerns included participants who had themselves acted as managers but reported trying to approach things differently:

value is attached to what’s easier for the system, what’s easier in terms of it being reliable and easy to describe and we’ll all do the same thing, so it’s easier to teach, versus the value of truly understanding what value is and how you add it … the NHS is a really system‐focussed organisation, and even when it’s good for its patients it’s still focussed on what it needs for itself and what it has to report for itself.

(Participant 16)

But I think it’s probably about a focus on people and meeting the needs of people as opposed to a focus on the system and the business of the system and meeting the needs of the system, which feels like it still trumps … it looms too large still, that one, it needs to be damped down.

(Participant 20)

Participants who represented patient perspectives or worked closely with service user communities often stressed the importance of breaking down or ‘undoing’ institutional thinking and boundaries, including by recognising that improvements can originate from users and from movements outside of healthcare institutions:

The idea of improvement needs to embrace different sorts of outcomes, like improvements in relationships, in what matters, so I think you have a series of narrowing lenses on what an improvement activity can achieve. So, if you exclude people like me from the room, you’re already narrowed on an institutional fix.

(Participant 15)

Well, shared decision‐making is a thing, it’s a thing because people aren’t trained in the first place to have a dialogue with the person they’re treating, about options and things. … I see quite a lot of modern stuff as being about trying to undo some of the distance travelled. I just think that shared decision‐making ought not to be a thing.

(Participant 11)

some of the most innovative thinking, in my opinion, comes from people as service users … putting it crudely, the more we have chairs with user experience, the more there are PhDs undertaken by people that are service users, the more careers, the more journal articles, the more journals that include people as service users in their reviewing, we are making progress.

(Participant 14)

So much of the improvement science work is embedded in all kinds of, you know, governance and structures and formal organisational processes … it’s almost been to the complete neglect of the kind of change, wider change, that can be driven from outside organisations.

(Participant 6)

### Perspectivalism within improvement research

Of course, being an academic or researcher is as much a role position as is being a service leader, manager, clinician, patient or service user. Furthermore, in an inherently applied field research, scientists have to consciously work with and navigate across groups of people operating from these various vantage points. Participants’ accounts illustrated the social embeddedness of their identities as they negotiated practical and epistemic tensions.

Participants, including academic participants, often resisted applying ‘research field’ labels to themselves but the most commonly adopted ones were ‘improvement science’ and ‘implementation science’. In relation to this distinction, both contrasts and commonalities were stressed, sometimes by the same participants. A few of the participants emphasised the increasing convergence of these currents whilst noting that building joint networks and shared community across this historical divide was much easier in some instances and climates than others. These discussions illustrate a ‘local’ example of what Knorr‐Cetina (2007) characterises as the ‘disunity of science’:

Implementation science might be about getting something, anything, an intervention into practice; improvement science is much more about the science behind how you get something better, so that’s about theories, methods, measurement, evaluation. Now, I think some of the same principles were there for implementation science but it was definitely a kind of a case of saying, no, we are different. And I think the two fields would say we’re linked but we are different.

(Participant 2)

So those two people, those two groups, do not come together to work together because they kind of feel like they’re divergent paradigmatically or methodologically or worldview—all of those things.

(Participant 1)

There’s paper after paper emerging as sort of comparison between improvement and implementation sciences. And I think the reality is you can say that they’ve started from different starting points … they’ve had different trajectories.

(Participant 7)

Researchers sit within currents with different histories and orientations. In this case, for instance, they broadly reflect origins on the one hand in reviewing and refining organisational processes (improvement science) and on the other hand in the identification, application and spread of evidence‐based interventions (implementation science). But there are many other overlapping variables in play in the constitution of research approaches and emphases. These include, for example, differences in terminology, problem framing, methodology and aims as well as in assumptions about who one is working for and with (as discussed in the previous section). Here, again various dichotomies feature in the participants accounts. As is notorious in applied social research, this includes the contrast between qualitative and quantitative methodological approaches. But this distinction sometimes appears alongside other distinctions—human/technical or cultural/structural etc.—which are loosely, and always contentiously, related:

Healthcare … is all about human interaction … And I think a lot of science ignores that or kind of puts it somewhere else. And I think very, sort of, quantitative measurement projects, and some of which I’ve described, kind of almost reduce it to something that it’s not or take it out of the equation.

(Participant 1)

So, if what you’re trying to sort out is hospital flow, waiting times, the kind of bottleneck sorts of things, then it makes sense to be using the process‐mapping quality, you know, the kind of quality control type approach, like, you know, your Virginia Mason type approaches. If what you’ve got is a cultural problem, it doesn’t matter how much process‐mapping you do, the culture will always undermine all the structural changes you put in place.

(Participant 18)

[On professional development courses] you would start often by saying, well, you know, improvement is 80 percent about people and relationships and 20 percent about technical skills, nominally. And then we would go on to spend 90 percent of our time talking about technical skills.

(Participant 20)

The interview data highlight that debates about paradigms and their comparative strengths and weaknesses are often not ‘detached’ scholarly questions but are questions about what kinds of knowledge have currency within dominant epistemic communities in a working context heavily shaped by biomedical norms:

within health research as a whole, we have a biomedical model of thinking about research, and methodology that’s followed on from that. So, you know, people value research that has a clinical effectiveness endpoint at some point.

(Participant 7)

So, when you think about measurement, which is an integral part of quality improvement, people will generally gravitate towards those parameters that lend themselves easier to measurement i.e., process/systems/procedures, whereas concepts like experience, for example, it’s qualitative and the way we’ve organised ourselves is that quantitative measurements take pride of place over qualitative. And so, parameters that fall within the qualitative bucket don’t tend to be as attractive to focus on.

(Participant 18)

Given that health care has an indefinitely large number of aspects, there are countless starting points for improvement and epistemological and ontological frames for research. Starting points matter because much follows from—is prefigured in—one’s starting point, something we will come back to in the discussion section.

Two participants explicitly cited the idea that ‘if your only tool is a hammer then all your problems have to be nails’. In addition, a few participants highlighted the danger of researchers being captured by particular models of inquiry rather than consciously embracing a more open‐ended conception of research along with some disciplinary eclecticism:

So, there’s a tension between the kind of core methodology that you try and apply to everything, … trying to force it into a … model that doesn’t help at all. And this is not helped by the improvement journals, you know, demanding that you write it up in PDSA format [and] … using a variety of methods and approaches according to the problem you’re tackling, which is like a research view.

(Participant 19)

I think you need to be as good at social psychology as you are at mathematics and probability, and you have to understand that both of those are disciplines that people spend their whole lives exploring and you have to draw down on their expertise and synthesise it … because it’s where they intersect that you become a more sophisticated improvement practitioner.

(Participant 9)

Arguably underpinning, or at least reinforcing, epistemological and methodological diversity is the disciplinary and interdisciplinary identities that several participants reflected upon. This included the idea that both the kinds and the amounts of theorisation researchers deployed can be a function of immersion in disciplines. Participants’ accounts also suggested that problem definition and thus the implicit framing of ‘improvement priorities’ can depend on disciplinary lenses. This emerged, in particular, in discussions of contrasts between psychology and sociology:

one of the key unresolved tensions that keeps coming up is, you know, an apparent perspective focussing on the system and organisational issues, or perspectives that focus on individuals. … You know, what comes first, does culture come before behaviour or does behaviour come before culture?

… to some extent it also reflects disciplinary perspectives, so a lot of the more individual behaviour people are probably coming from, you know, behavioural perspectives, you know in my case a sort of more clinical or health services research perspectives, and the organisation people often are more policy or sociological.

(Participant 12)

We’ve got our solutions, we’ve also got our fads and fashions and we’ve got our own mindsets that drive us in certain directions. … I’m a sociologist so I’m going to start at the opposite end so I’m going to come at everything from how do I explain this phenomena as a consequence of a social, cultural way of doing stuff and not be that concerned about individual psychology.

(Participant 2)

### Partial knowledge: Expertise as scepticism and critical reflexivity

And one of the things that I’ve tried to think about is why improvement doesn’t work, like why doesn’t it work actually most of the time. …that’s not a rare occurrence, that’s a common occurrence.

(Participant 9)

Participants spoke quite frequently and freely about the limitations of improvement expertise and its tendency to be ‘partial’ in one way or another. Their accounts included a good deal of scepticism about improvement expertise. In an expanding field, this makes sense. As expertise about improvement expands—incorporating more improvement problems and agendas, more models, tools, disciplinary resources and more improvement actors and vantage points, then so also will internal debate and critique. Diverse and sometimes competing sources of expertise inevitably lead to epistemic contests. And, of course, contests about knowledge are always, at the same time, contests about circulations of power: ‘there is no power relation without the correlative constitution of a field of knowledge, nor any knowledge that does not presuppose and constitute at the same time, power relations’ (Foucault, 1977, p. 27).

In a sense, the whole field of improvement is rooted in scepticism. It is based on questioning the way health‐care services are organised and enacted. The rise of improvement science arguably represents a further stage of scepticism based on questioning the knowledge bases of improvement practices. Finally, the contests and uncertainties within the academic field of improvement research can be seen as a further, higher‐order and reflexive scepticism about (aspects of) improvement and implementation sciences. A large proportion of the ground covered above can be placed in the category of scepticism. As we have discussed, participants often articulated what they were aiming to do by distinguishing it from the various ways in which claims to knowledge can be inadequate or even misguided. For example, by being reductionist (perhaps overly biomedical, technicist or ‘top‐down’); by being too fragmentary, or characterising problems too broadly; by being out of touch with practice or too embedded in the taken for granted of practice; by neglecting the perspectives of patients (or clinicians) and so on.

Even where participants were concerned about the limitations of the existing state of, or promise of, health‐care improvement their critical reflexivity could be seen as epistemically valuable as a check on over‐claiming and as a support to intellectual humility. In addition, one relatively optimistic theme that arose from the interview data was the possibility of moving beyond ‘fragmented knowledge(s)’ towards various kinds of syntheses. On the one hand, participants often underlined the limited lenses—on both health care and quality—deployed in improvement knowledge production:

I think you get, like anywhere, you get discrete pieces of work in discrete sectors or silos or professional groups … but yet it’s very difficult—I understand that’s why it’s not done very much—it’s very difficult to get someone looking across a whole system or a whole hospital.

(Participant 1)

And, for example, people might do a, sort of, a cost‐effectiveness or a cost‐saving analysis, and that’s a study in its own right and that’s only one dimension of many. So I don’t think you ever see studies where all of the dimensions of quality are actually evaluated.

(Participant 7)

On the other hand, participants pointed to ways of potentially ‘overcoming’ these limitations by ‘knowledge integration’—pulling together different standpoints, perspectives and disciplines:

there’s stuff that needs to be done at the local level but you need to recognise that there’s all these system factors around it, and the complexity of the system, that impacts on how that actually works, … that transdisciplinary kind of multi perspective.

(Participant 2)

And so part of the challenge, I think, is to foster this kind of shared understanding of what works: top‐down/bottom‐up.

I think that the key here is the integrative knowledge that in fact is required to link the knowledge of the lived reality of the person … the science‐informed practice and the lived reality of the … professional. The key for an improver is integrative thinking …

(Participant 3)

So our work is horizontal, transversal, across vertical programs.

I think it’s really necessary to have these communities talking together, people working more on the conceptual framework and people with this objective of providing services in the field. But we really need to have this dialogue, I think, between the two communities.

(Participant 4)

Some of the scepticism expressed by participants was very hard‐hitting—suggesting that the field of health‐care improvement has failed to make a big enough difference or ask big enough questions. The two most common, and often explicitly linked, concerns were about whether health‐care improvement has sufficiently attended to the potential marginality of service users or to the persistence of health inequalities—in other words, questions about power differentials and structural disadvantage. Participants with strong links to patient organisations summarised their perceptions of some health‐care colleagues with this emphasis:

I mean, I still see it like that, that the world of quality improvement seems to be more or less free of any insights from the social sciences other than perhaps some psychological ones, I don’t know. But certainly they never talk about power and … it’s not really … reflected in wanting to learn from the social sciences.

(Participant 11)

the kind of people who’ve invented it as a school of thought. …

doesn’t really lend itself to ask questions about power and voice and experience and inequality, and the “so what?”. You know, why are we trying to make healthcare better? …

But when I see the kind of projects these people lead on and speak of with pride, I think they are so poxy, you know, like because you haven’t asked the question of power, … you’re going to improve the so‐and‐so thing about your … pathway and you haven’t really reflected on the fact of how disempowered you are in your organisation and how really there’s so much that needs changing … I’m going to measure this and then I’m going to measure it again. And, lo and behold, the number has changed. So what?

(Participant 13)

It is worth stressing that similar critiques were also made by other participants. This included articulations of the importance and relative neglect of power relations and politics:

you’ve got these big, dominant perspectives that are still shaping the field … We [sociologists] have to be there at the table but it’s just always that, “Oh, it’s that bloody sociologist again, going on about politics and power.

(Participant 2)

we aren’t that different than when Hippocrates or whoever it was first said it, which is that if most of what you do is of no benefit at least make sure that you’re not giving harmful things to people … yet we all live our lives based on a philosophy, the neoliberal one, that selfishness and market competition will be the answer to everything, which has clearly not proven to be true, and to be patently false by all of the, I mean, massive inequalities.

(Participant 17)

Even participants who unequivocally identify with the academic and health services improvement communities, whilst emphasising the value of improvement contributions were often keen to question the way priorities are constructed:

we need to rebalance some of those metrics and say, well, actually it doesn’t really matter if you’ve got a brilliant effectiveness treatment that works for a tiny minority of patients that you can reach and yet you’ve got, you know, hundreds of patients, for example some communities or sectors of the population that are completely shut out of this.

(Participant 7)

we reduced catheter‐associated urinary tract infections or we reduced pressure ulcers or we reduced fall‐related injuries. I mean, those are…they’re, in any clinical context, each of those targets is one of about 50 that you could have picked. And … …it isn’t even necessarily the case that they’re the most important target… So, the actual, you know, impact is small, very small. So, I don’t really know what to say except that we only ever make progress that is really, really marginal.

(Participant 17)

The potential neglect, or even exacerbation, of health inequalities came up regularly in the context of these sceptical remarks about improvement priorities:

they will often choose to focus improvement activity on tried and tested areas. Diabetes is a really common one. You know, it’s relatively easy, it’s relatively straightforward …. How often do improvement programs focus specifically on health inequalities and improving those who have greatest need? Again, not that often.

(Participant 5)

not addressing the needs of the most vulnerable, marginalised, disadvantaged is, I think, a big problem … And so the risk is always that you’re … making the marginally disadvantaged citizens even more marginal and disadvantaged from the process.

(Participant 6)

## DISCUSSION

Judged by volume of activity, including the proliferation of academic journal titles, health‐care improvement knowledge production has substantially increased over the last 25 years (Scaccia & Scott, 2021; Sun et al., 2014). Sets of improvement‐related studies, practices and discourses are now widespread in academic and health services contexts. The ambition to build and strengthen improvement expertise should be welcomed because improving health care, including tackling shortcomings and strengthening provision is both practically and ethically important. However, it is also necessary to acknowledge relevant complications and qualifications, many of which are made evident in the findings. In summary, participants’ accounts illustrate the prevalence within the field of (a) multiple perspectives and voices, (b) a considerable capacity for critical reflexivity and scepticism and (c) concerns that particular ‘ways of knowing’ are nonetheless relatively privileged.

The expertise of the participants was manifest in their capacity to take a second‐order view of the field. As their accounts make clear ‘more’ knowledge is not simply more of the same. The field is evolving and dynamic and this means that conceptions of improvement knowledge and practice deriving from different periods and orientations sit beside one another. In addition, participants frequently spoke about the relative privileging of quantitative over qualitative ways of knowing and some also elaborated on what they saw as the comparative centrality and marginality of different disciplinary paradigms and lenses. This self‐consciousness about contestation feeds into and underlines the increasing relevance of sociology in the field. Sociology is now widely accepted as an important ‘feeder discipline’—that is, in work that might be crudely labelled as ‘sociology *for* improvement’. Some of the foremost improvement scholars draw heavily upon their sociological expertise in influential policy and practice contributions. At minimum, sociological agendas and lenses strengthen the repertoire, analytical power and rigour of more conventional or ‘orthodox’ improvement paradigms by surfacing value assumptions and power relations (Waring et al., 2016, p. 204).

Furthermore, there are no clear distinctions between sociology ‘*of*’ and ‘*for*’—sociological work that analyses and critiques improvement as a field, both of practice and research, can inform debates in improvement and thereby also has the potential to valuably influence or inflect practice (Cribb, 2018). The issues highlighted by the interviews are amenable to critical sociological analyses, many elements of which are indicated or articulated by the participants themselves. Here, we can just touch on a few of these. In particular, we wish to underline the risks of dominant approaches to health‐care improvement knowledge production ‘amplifying’ the influence of contested norms, here including norms related to what counts as ‘quality’ or ‘good health care’ and norms related to appropriate approaches to ‘doing improvement’. This threat of amplification follows, in part, simply because improvement projects encompass numbers of people, especially if we take seriously the ‘scaling up’ or ‘spread’ aspirations common within the field. But it also, of course, reflects the core conceptual and practical function of improvement which involves identifying, labelling—and also promoting and pursuing—particular conceptions of ‘quality’. In this regard, health‐care improvement is strongly normative in a jointly philosophical and sociological sense, that is, it is necessarily value laden, embodying ethical stances, and it promotes, reinforces and ‘polices for’ particular norms over others.

Many participants—in a way that cut across role positions—emphasised the tendency for health services to privilege and reinforce ‘ways of knowing’ that are salient to systems, especially managerially defined conceptions of institutional success. This carries the threat of relative neglect to other perspectives. The growth of improvement expertise as a field might be understood as about the possibility of establishing forms of authority about ‘good health care’ that are independent of simple system reproduction—whether, for example, these are founded on scholarship and/or extra‐health‐system vantage points. In this context, appeals to expertise, including improvement research or ‘science’, can play a significant function as a complement to, or potentially powerful counterweight to, system norms. However, quite a few participants, not least those representing patient perspectives, presented not only institutional and managerialist logics but also other currents of improvement knowledge as a stumbling block to much needed change. One version of ‘improvement’—biomedically technicist (or ‘nerdy’ in one characterisation)—is here constructed as the problem that needs to be tackled by other readings of what improvement and improvement knowledge can and should mean.

In broad terms, the norms highlighted and subjected to questioning by many of our participants are familiar ones being regularly subjected to critique across sociology of health. These include undue weight being placed on technocratic and measurement‐driven processes including managerialism; the institutionalisation of biomedical power, positivistic conceptions of evidence combined with strong knowledge hierarchies; and reductionist framings of health, persons and what counts as ‘success’ (Holmes et al., 2006; Lupton, 2012). More broadly, these are continuous with a long tradition within sociology of critiquing the power of medical knowledge in controlling or marginalising other perspectives—including the classic work of, for example, Illich (1976) and Freidson (1970)—a tradition whose central relevance to health‐care improvement has been noted (Waring et al., 2016, p. 205). In one sense, improvement policy and practice simply represents another set of sites to analyse in this way. However, we are suggesting that improvement expertise is itself worth investigating as one mode through which such norms can be reproduced and amplified across an indefinitely wide range of sites. Amplification processes can be defended (as reinforcing and disseminating good practice) or seen as processes that need to be resisted, interrupted or at least questioned and moderated.

The diversification of what is taken seriously as improvement expertise is potentially an invaluable resource for health services. It means that in addition to the unquestionably important participation of a range of voices and perspectives in improvement practices, there is a place for foundational disagreements and challenges about improvement foci, purposes and approaches. However, forms of improvement expertise do not come together as equals. From the accounts of the participants, there are seemingly pressures in two directions—a welcomed growth in diversity of perspectives and knowledge resources accompanied by continuing processes that can diminish the impact of this diversity by reinforcing knowledge and power hierarchies. The combination of health system and positivistic logics, especially where they reinforce one another in quantitative and ‘what works’ models, can effectively attenuate the influence of other lenses which are at the same time judged necessary for understanding and enacting improvement. This requires consideration by those concerned about health‐care improvement amplifying technicist and biomedical norms. In particular, it requires attention to what Knorr‐Cetina calls ‘the interiorised processes of knowledge creation’ (Knorr‐Cetina, 2007).

Boulton et al. (2020) offer considerable insight into these processes in relation to implementation science. On the one hand, they set out how implementation science has been successful in broadening the conception of the evidence base in health care. This has included self‐consciously drawing from a broad range of social science perspectives in ways that could potentially reconfigure ‘the balance between methodologies and epistemologies in healthcare’ (p. 380). Yet, on the other hand, they lament the relative absence of this reconfiguration, arguing that there has been insufficient focus on the ‘productive’ aspects of these broader methodologies and also on their ‘irreconcilability’. On their account, methodological diversity appears on the surface but risks to a large extent being cancelled out. Although the epistemological repertoire is nominally broad, the tendency is for disciplinary and theoretical diversity to be shrunk into descriptive assemblages largely geared towards ‘what works’ prescriptions. One of the mechanisms that enables this, they suggest, is a reliance on using ‘middle range’ theories as tools in ways that uproot them from the disciplinary and inter‐disciplinary contexts in which they are forged and fought over. The result is that the value of critical perspectives is lost or diluted and that ‘the concept of “knowledge” or “science” used in these titles may not reflect the ways in which “knowledge” or “science” are conceptualised as contextual, contested and contradictory in other social science methodologies.’ (p. 382).

Closely analogous concerns have been expressed by other scholars writing about improvement and such concerns suggest that there are limits to the extent to which ‘integration’ should be thought of as an answer, or at least a ‘tidy’ answer, to knowledge fragmentation. Participants in this study spoke about the value of substantive integration—that is, bringing together analyses of different parts of systems or institutions and attention to different aspects of quality. Yet not everything can be tidily brought together. Whilst welcoming many efforts towards substantive integration, we should be more cautious about the notion of epistemological integration. Ways of knowing can pull in different directions and that need not be viewed exclusively as a problem but rather as enlarging the field, acknowledging inherent tensions and even as enabling practical creativity. Sociological contributions to quality improvement agendas, it has been argued (Zuiderent‐Jerak et al., 2009), should not be judged against narrow conceptions of ‘usefulness’—especially where this means simply discovering ‘what works’ to solve a pre‐defined quality or safety agenda. Rather, these authors suggest, this emphasis ‘may undo one of the strongest assets of good social science research: the capacity to complexify the taken‐for‐granted conceptualisations of the object of study’ (p. 1713). This alternative conception of ‘usefulness’ not only allows for the theoretical analysis of ‘multiple ontologies’ but also helps opens up alternative possibilities for ‘acting with’ quality improvement agendas.

A case has also been made to move away from the passive, tame use of ‘theories’ as tools towards harnessing the power of ‘theorising’ in improvement work. Theorising in this active creative sense can include both reflecting on epistemological and ontological contradictions and helping to rethink and reconstruct frames, concepts and practices (Kislov et al., 2019). We suggest that work on the sociology of improvement expertise—building on the findings and analyses offered in this article—may make a contribution to this effort. Eyal (2013) has argued that the power of expertise (in contrast to traditional accounts of professional power) can operate through inclusion and ‘generosity’ rather than ‘monopoly’ and ‘autonomy’. Only by extending and sharing knowledge networks that span multiple social fields and kinds of actors can experts elicit forms of cooperation and influence. To the extent that these dynamics are understood and made explicit then there is scope to self‐consciously affirm, rework or interrupt them. This contribution could even be led by sociologically informed improvement researchers, perhaps working in conjunction with reflective system leaders and policy makers, with the aim of surfacing and deliberately working on epistemic tensions and processes of co‐option in the field (cf Rycroft‐Malone et al., 2016). In some ways this would be analogous—but at a meta‐level—to the investment that, in the best instances, goes into co‐production at the level of practice (e.g. Donetto et al., 2015).

Two questions that need such consideration are as follows: First, what range of things might improvement expertise involve if we were to start somewhere quite different from an identified ‘performance gap’ or ‘implementation gap’ (Nilsen et al., 2022) and especially if our starting points place centre stage the challenges and opportunities for improvement that arise from embracing contestation about both the ends and means of improvement? This includes recognising power imbalances, structural inequalities and the multiplicity of relevant agents, including forms of agency and activity located outside health services that draw, for example, on social movements and community resources. Second, how far does it even make sense to try and assimilate these more radical and far ranging agendas into the domain of improvement expertise? These agendas not only suggest different constructions of quality and improvement but also alternative foci and approaches to both research and practice. As we have noted the ‘machineries of knowing’ captured in the accounts of many of our participants have the potential to reinforce and amplify norms about which these same participants are sceptical. Given this, it may be wiser simply to welcome improvement‐related discourses and practices continuing to evolve into sets of contradictory and complementary strands rather than to aim for greater consolidation. A field which embraces divergence and disagreement about the nature and value of knowledge claims may have advantages for all. This includes advantages for those who champion the value of more conventional, even ‘technocratic’, improvement expertise. There needs to be space to highlight existing successes and to defend the importance of that contribution, which reflexive critique qualifies rather than erases.

In conclusion, in the domain of health‐care improvement, there is no single coherent and stable ‘epistemic community’, but rather a set of overlapping communities. And such communities do not produce ‘neutral units’ of knowledge; they ‘“act with” knowledge. They produce, publicise and police knowledge; … They change and are situated on trajectories and thus articulate histories, futures and possibilities’ (Meyer & Molyneux‐Hodgson, 2010). The participants in this study offered expert insights into how improvement knowledge production creates and constrains the identities of knowledge producers and shapes what counts as the objects of knowledge.

## AUTHOR CONTRIBUTIONS

**Alan Cribb**: Conceptualization (Lead); Data curation (Equal); Formal analysis (Equal); Funding acquisition (Lead); Investigation (Equal); Methodology (Equal); Project administration (Lead); Writing – original draft (Lead); Writing – review & editing (Equal). **Vikki Entwistle**: Conceptualization (Supporting); Data curation (Equal); Formal analysis (Equal); Funding acquisition (Equal); Investigation (Equal); Methodology (Equal); Project administration (Equal); Writing – original draft (Supporting); Writing – review & editing (Equal). **Polly Mitchell**: Conceptualization (Supporting); Data curation (Equal); Formal analysis (Equal); Funding acquisition (Supporting); Investigation (Equal); Methodology (Equal); Writing – original draft (Supporting); Writing – review & editing (Equal).

## Data Availability

The data that support the findings of this study are not publicly available due to privacy or ethical restrictions.
